# Clinical significance of fluid biomarkers in Alzheimer’s Disease

**DOI:** 10.1007/s43440-020-00107-0

**Published:** 2020-05-08

**Authors:** Piotr Lewczuk, Marta Łukaszewicz-Zając, Piotr Mroczko, Johannes Kornhuber

**Affiliations:** 1 Lab for Clinical Neurochemistry and Neurochemical Dementia Diagnostics, Department of Psychiatry and Psychotherapy, Universitätsklinikum Erlangen, Friedrich-Alexander Universität Erlangen-Nürnberg, Schwabachanlage 6, 91054 Erlangen, Germany; 2 Department of Neurodegeneration Diagnostics, Medical University of Białystok, Białystok, Poland; 3 Department of Biochemical Diagnostics, Medical University of Białystok, Białystok, Poland; 4 Department of Criminal Law and Criminology, Faculty of Law, University of Białystok, Białystok, Poland

**Keywords:** Alzheimer's Disease, Biomarkers, Cerebrospinal fluid, Amyloid, Tau

## Abstract

**Abstract:**

The number of patients with Alzheimer’s Disease (AD) and other types of dementia disorders has drastically increased over the last decades. AD is a complex progressive neurodegenerative disease affecting about 14 million patients in Europe and the United States. The hallmarks of this disease are neurotic plaques consist of the Amyloid-β peptide (Aβ) and neurofibrillary tangles (NFTs) formed of hyperphosphorylated Tau protein (pTau). Currently, four CSF biomarkers: Amyloid beta 42 (Aβ42), Aβ42/40 ratio, Tau protein, and Tau phosphorylated at threonine 181 (pTau181) have been indicated as core neurochemical AD biomarkers. However, the identification of additional fluid biomarkers, useful in the prognosis, risk stratification, and monitoring of drug response is sorely needed to better understand the complex heterogeneity of AD pathology as well as to improve diagnosis of patients with the disease. Several novel biomarkers have been extensively investigated, and their utility must be proved and eventually integrated into guidelines for use in clinical practice. This paper presents the research and development of CSF and blood biomarkers for AD as well as their potential clinical significance.

**Graphic abstract:**

Upper panel: Aβ peptides are released from transmembrane Amyloid Precursor Protein (APP) under physiological conditions (blue arrow). In AD, however, pathologic accumulation of Aβ monomers leads to their accumulation in plaques (red arrow). This is reflected in decreased concentration of Aβ1-42 and decreased Aβ42/40 concentration ratio in the CSF. Lower panel: Phosphorylated Tau molecules maintain axonal structures; hyperphosphorylation of Tau (red arrow) in AD leads to degeneration of axons, and release of pTau molecules, which then accumulate in neurofibrillary tangles. This process is reflected by increased concentrations of Tau and pTau in the CSF. 
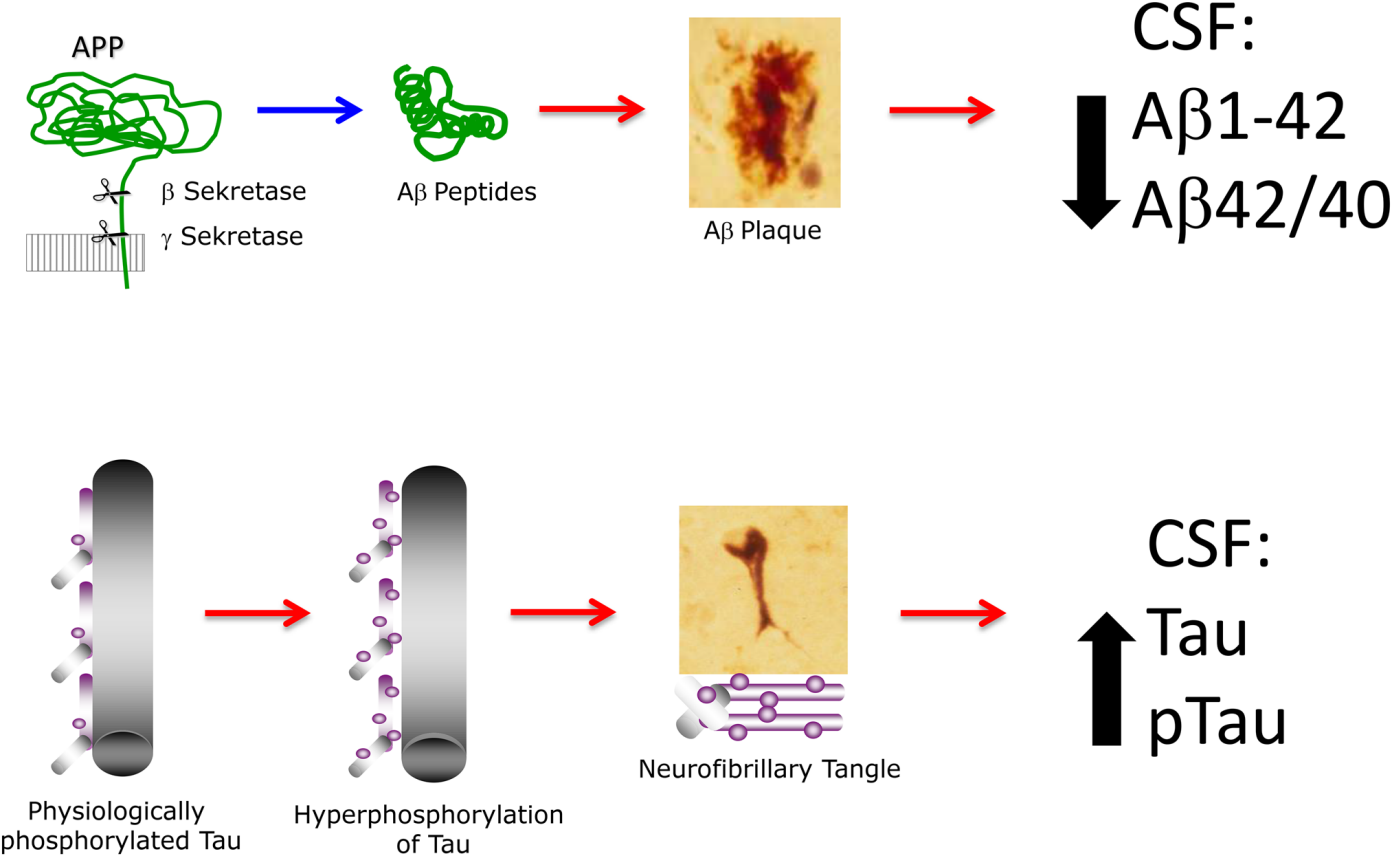

## Introduction

Alzheimer’s Disease (AD), a complex neurodegenerative disease, is characterized by progressive cognitive impairment to the extend impacting activities of daily living, such as episodic memory loss and alterations in spatial and temporal orientation. The disease is the most common cause of dementia and cognitive decline in subjects over 65 years of age [1]. AD is a growing global public health priority concerns with serious implications for society. It is a condition of mature population, usually doubling in prevalence after age of 65 every 5 years [2, 3]. Currently about 14 million patients in the USA and Europe are afflicted by AD, and 40% of this population is over the age of 85 [4, 5]. The total costs of this disease and other types of dementia associated with health care, long-term care and hospice were estimated at $290 billion in 2019 in the United States only. Moreover, the AD incidence increases with age, thus its prevalence is rising due to ageing of population [3].

Interestingly, European epidemiologic studies show that dementia prevalence was stable from the late 1980s to the early 2000s, which, taken together with increased survival of patients, suggests that incidence of dementia may have decreased during this time interval [6, 7]. It is hypothesized that general lifestyle improvements: early recognition and treatment of diabetes and hypertension (two important risk factors for development of dementia), as well as reduction in prevalence of cigarette smoking might be responsible for this trend [8].

It has been estimated that about 44 million people live with dementia worldwide and this number may triple by 2050 due to the population ageing. The highest increase in the prevalence of dementia is predicted in middle- and low-income countries [4, 9].

Pathologic alterations of AD start in medial temporal lobe and then afflict the areas of neocortex [10, 11]; the changes beginning decades before the onset of the clinical symptoms [3, 12]. AD progresses throughout three stages of pre-symptomatic stage, prodromal stage, such as mild cognitive symptoms, and eventually a symptomatic stage with dementia [13]. Additionally, the MCI (mild cognitive impairment) stage predicts the cognitive dysfunctions of dementia. Approximately 10–20% of MCI patients converted to AD every year [14].

The clinical symptoms is usually preceded by preclinical phase (mainly symptoms-free), thus early diagnosis of AD remains difficult [13, 15, 16]. AD biomarkers are usually tested when patient has already progressed to the MCI (or later) stage. It has been proved that assessment of cerebrospinal fluid (CSF) may reasonably predict progression of MCI to AD with accuracy of above 80% [17–19].

Pathophysiology of AD relays on amyloid beta (Aβ) plaque accumulation and neurofibrillary tangles formed by Tau fibrils as well as neuron and synaptic degeneration, neuroinflammation and glial activation. Extracellular senile plaques, consisting of Aβ peptides and intracellular neurofibrillary tangles, composed of mainly hyperphosphorylated form of Tau (pTau) proteins were proved to be neuropathological features commonly presented in the brains of AD patients [20]. Thus, these two groups of molecules were found to be the best-validated AD biomarkers.

## Cerebrospinal fluid and lumbar puncture: medical and legal perspective

CSF is generated in the ventricles of the brain, from where it flows around the brain hemispheres, and along the spinal cord, to be ultimately absorbed back to the blood. Since it stays in the direct contact with the brain tissue, it is enormously important source of diagnostic information about physiologic and pathologic processes in the central nervous system (CNS). On the other hand, along the CSF flow path blood proteins diffuse passively into it according to the concentration gradient. This diffusion is known as the blood–CSF barrier (not to be mixed with the blood–brain barrier). Hence, a CSF sample, in most cases collected by lumbar puncture (LP), contains a mixture of proteins originating from the CNS and from the blood. Since it is exclusively produced by the liver and enters the CSF exclusively by passive diffusion, its CSF/serum concentration quotient (Q_Alb_) is commonly accepted biomarker of the blood–CSF barrier status. Many other blood proteins, like immunoglobulins (Ig’s) diffuse passively to the CSF under normal conditions, but under pathologic conditions, known as neuroinflammation(s), they are also generated in the brain. Since CSF stays in a direct contact to the brain tissue, it is obviously ideal source of biomarkers of brain disorders, like AD and similar neurodegenerative conditions [16].

Lumbar puncture is a routine medical procedure for the collection of CSF for diagnostic purpose. Each medical activity is regulated by the legal provisions of the country in which it is carried out. This requires physicians to know the basic principles for conducting medical procedures resulting from the legal regulations of a given country. Medical law is different in various jurisdictions, but among the many important provisions that apply to doctors in various countries around the world, one can point out the chief issue, which is of particular importance. This is the legal regulation of the patient's informed consent for medical treatment, such as LPs. The importance of this problem is evidenced by the fact, that in some countries the issue of a patient's decision regarding his medical treatment is regulated in a separate legal act. An example of such a solution is the legal system of England and Wales, where legal regulations regarding this issue are included in the Mental Capacity Act 2005.

This is an extremely important issue regarding the physician’s relationship with the patient, because it defines the limits of the rights of the person performing medical activities towards the patient and indicates the basic duties of the physician during the treatment process. On the other hand, the right to consciously decide on treatment protects the patient's basic interests and clearly defines the rights he is entitled to. In various jurisdictions, it is a rule that all medical procedures as well as diagnostic or therapeutic activities require patient consent. This also applies to conducting LPs. This principle may have a different background, depending on the legal culture of the country. It results in the patient's participation in the treatment process. This right is directly related to the physician's duty to inform the patient about his health. Only in such a situation can a person without medical education be able to consciously decide about their treatment.

However, in some situations it is not possible to express patient’s informed and voluntary consent. The legal systems of different countries distinguish specific categories of patients who are unable to make an informed decision. This applies among others to people who, due to various types of mental disorders, are recognized by law as being unable to give consent for medical intervention. Although the procedure for determining that a patient is not able to make a decision about their treatment is different in different countries, generally the decision on this case is taken by the court. In such cases, legal provisions designate other people who make such decisions on behalf of the patient. Depending on the situation, these can be parents, legal guardians or for example a person's guardian. Certainly, there are some differences between the legal provisions regarding giving informed consent for medical procedures in different countries, which result, among others, from historical issues [21, 22].

## Aβ peptides and Aβ oligomers

Aβ peptides are main component of senile plaques, derive via the enzymatic cleavage of β-amyloid precursor protein (APP) [23]. Thus, Aβ is formed by the sequential cutting of APP via β-site amyloid precursor protein-cleaving enzyme 1 (BACE1) as well as γ-secretase. Subsequently several forms of Aβ peptides are released [24]. In addition, APP might be processed in non-amyloidogenic pathway by α-secretase, which leads to the release of soluble APPα. Cleavage of APP at different positions by the γ-secretase leads to release of Aβ peptides of variable length [25].

The 42-amino acid isoform of Aβ42 peptide accounts for not more than 5–10% of the total Aβ peptides in the human CSF [26]. However, Aβ42 is a major component of the plaques in the AD patient brains [27, 28]. Investigations have indicated that the CSF levels of Aβ42 correlate inversely with plaque load, what was found in autopsies or/and in vivo with positron emission tomography (PET) [29, 30]. The mechanisms leading to the decreased concentrations of Aβ42 in the CSF of AD patients are still unclear. Some authors indicated that it might be a result of the Aβ42 sequestration in AD plaques with reduced availability to be cleared into the CSF [31]. However, reduced CSF Aβ42 concentrations may be also found in other diseases, without plaques, like bacterial meningitis [32]. Thus, presented theory doesn not explain a selective lowering in the CSF Aβ42 concentration [33]. Other hypothesis includes reduction in the rate of Aβ42 generation [34] or increased degradation of Aβ42 [35].

The decrease of CSF Aβ42 concentrations in AD patients has been validated in many studies that proved consistent findings concerning a mean fold change of 0.56 for CSF Aβ42 compared to cognitively unimpaired elderly [36]. The study of Hulstaert et al. [37] has estimated that the sensitivity and specificity of the use of Aβ42 alone to distinguish AD patients from elderly controls were 78% and 81%, respectively. Consistent findings were evaluated by other authors, who also reported similar sensitivity and specificity (78% and 83%, respectively) in the differentiation between AD patients and elderly controls [38]. It has been estimated that the CSF Aβ42 measurement is useful in the correct classification of 87% of the subjects, when non-Alzheimer’s dementia patients and non-demented individuals were compared [39]. It is also suggested that decrease of CSF Aβ42 is early indicator of clinically “silent” brain amyloidosis [40].

Despite CSF Aβ40 as alone biomarker is not promising AD biomarker, there is currently no doubt that the CSF concentration ratio of Aβ42/Aβ40 is superior to Aβ42 alone as a diagnostic tool for AD [15]. Several studies have shown its improved diagnostic accuracy compared with Aβ42 alone, or with other biomarkers [41–44]. The reason might be because Aβ42/40 compensates for abnormally high or low total Aβ load in the CSF in individuals, therefore normalizing inter-individual variability in CSF Aβ42 levels [43, 45].

Studies examining Aβ42/Aβ40 values as an informative tool across the spectrum of AD may be categorized into three main groups: (1) diagnostic studies for AD, including those using clinical diagnosis as reference (case–control design), and also including comparison to the amyloid PET as a proxy of AD pathology; (2) investigations of the differential diagnosis between AD and other neurodegenerative disorders; (3) prognostic studies, where the Aβ42/Aβ40 ratio was tested for its ability to predict progression from preclinical to the stage of dementia.

Concordance between Aβ PET imaging results and CSF biomarker concentration has been observed with different Aβ PET tracers. In some cases, studies of both AD patients and cognitively normal individuals showed an inverse, non-linear correlation between Aβ42 and amyloid PET, but not Aβ40, using the tracer Pittsburgh compound B (PiB) [29, 46]. Similarly, Aβ40 alone showed significantly lower discriminative power than Aβ42 alone in identifying ^18^F-flutemetamol-positive patients [47]. Some recent studies reported lower concentrations of CSF Aβ40 in PiB amyloid-positive individuals compared with a PiB-negative. While high concordance between CSF Aβ42 levels and amyloid-β PET imaging is now well established [48, 49], discordance between CSF Aβ42 levels and PET imaging-positive results is also a known phenomenon. Obviously, discordant results are more common in cognitively normal individuals [46, 50–52], which might be due to the two measures providing partially independent information [51]. However, the concordance with PiB imaging status clearly and highly significantly improves from ~ 75% for Aβ42 alone to ~ 90% for the Aβ42/Aβ40 ratio [30, 50]; CSF Aβ42/Aβ40 and Aβ42/Aβ38 ratios have also been strongly associated with ^18^F-flutemetamol PET status [53, 54].

The discordance seen in some investigations comparing Aβ42 in CSF and PET amyloid-β imaging leads to a hypothesis that they reflect different aspects of amyloid pathology [51]. Evidence that CSF Aβ42 concentration decreases before amyloid-β is detectable with PET imaging suggests that CSF Aβ42 is a more sensitive marker of AD at very early stages, while Aβ PET may be used for better grading of early AD [48]. Additional studies are needed to understand if discordant CSF Aβ42 and PET Aβ imaging could allow further stratification of preclinical AD patients [49]. Palmqvist et al. [55] compared CSF biomarker concentration with PET measurements in patients with MCI-AD rather than AD and found that amyloid PET and CSF biomarkers are able to identify early AD with high accuracy. They found no improvement when combining CSF and PET amyloid measures than using either CSF Aβ42 or tTau alone [55]. Hence, for the moment, the choice between using CSF or Aβ PET biomarkers for identifying early AD could be based on availability of PET scanners, the associated cost, and physician/patient preferences [56].

Biomarkers for AD such as the Aβ peptides have been found to have also some utility in helping to differentiate between AD and other types of neurologic conditions, such as non-AD dementia, which may have similar clinical symptoms [43, 57, 58]. Aβ40 levels were found to be lower in cerebral amyloid angiopathy (CAA) and CAA-related inflammation (CAA-I) [59, 60], FTD [61–65], vascular dementia (VaD), and dementia with Lewy bodies (DLB) [53, 58] compared with AD. Aβ42 levels often overlap between AD and other dementia groups with the exception of much lower levels observed in CAA patients [59]. Therefore, as a stand-alone measure, it is practically useless in differential diagnostics [61]. On the other hand, there is evidence that differences between AD and non-AD dementias may be more pronounced using a ratio of CSF Aβ42/Aβ40 (or Aβ42/Aβ38) than Aβ42 alone, and could therefore help differentiate AD from other disorders with similar clinical symptoms [50, 53]. The Aβ42/Aβ40 ratio has shown greater differential diagnostic accuracy compared with Aβ42 alone or other CSF biomarkers [43], with several studies applying Aβ42/Aβ40 ratio measures to distinguish AD from non-AD dementia [41, 53, 57, 58, 61, 65–71]. Examples of types of dementia in which the Aβ42/Aβ40 ratio has improved discrimination from AD and non-demented controls, compared with Aβ42 alone, are FTD [58, 61, 67, 72], VaD [58] and DLB [58].

Lewczuk and colleagues [41] demonstrated that the Aβ42/Aβ40 ratio identified more AD patients correctly from a population including AD, non-Alzheimer’s dementia (nAD) and control subjects compared with Aβ42 alone (AD vs controls: 94% vs 87% patients correctly classified; AD vs nAD plus controls: 91% vs 87%) [41]. The same conclusion was reached in another study from the same group, with entirely different cohorts, and different ELISAs [44]. Spies et al. [58] reported both sensitivity and specificity of > 80% using the Aβ42/ Aβ40 ratio to differentiate AD from FTD, DLB, VaD, and other non-AD neurodegeneration conditions. Together these studies strongly suggest that Aβ42/Aβ40 ratio may be also of use in differential diagnosis, provided the differential diagnosis question is properly formulated.

Aβ peptides longer than 42 or shorter than 40 amino acid residues have been also tested as potential AD biomarkers. For example, Aβ43 isomer has been reported decreased in AD, with a similar diagnostic accuracy as CSF Aβ42 [73, 74]. The clinical investigations have revealed no difference between CSF Aβ38 levels in AD subjects and control individuals [36], however CSF Aβ38 correlates with PET Aβ [75]. The authors conclude that the ratio of CSF Aβ42/Aβ38 is better at predicting Aβ-positive PET than CSF Aβ42 alone and comparable to CSF Aβ42/Aβ40 ratio [53]. Moreover, CSF Aβ42/Aβ38 ratio might be helpful in the differentiation between AD and dementia with Lewy bodies (DLB) [76] and other non-AD dementias [53].

Since oligomerization of Aβ monomers (mostly those ending at the C-terminal 42) seems the very first event on the pathway of the development of AD, Aβ oligomers seem to play a role in AD diagnostics. Soluble Aβ oligomers (AβOs) are more toxic than non-soluble forms in Aβ plaques [77]. It has been proved that AβOs isolated from brains of AD patients may reduce number of synapses and enhanced long-term synaptic depression in regions of brain, that are responsible for memory, what was presented on animal models of AD [78]. Some clinical investigations have revealed a significant increase in CSF AβOs in AD patients compared to age-matched controls. In addition, an inverse correlation between AβO levels and with mini-mental state examination (MMSE) score was observed. Based on area under ROC curve (AUC) of AβOs (AUC = 0.860 with 80% sensitivity and 88% specificity), these findings suggest the significance of oligomers in the diagnosis of AD patients [79]. Comparable results were demonstrated by other investigators, who assessed significantly elevated CSF AβOs concentrations as well as the AβOs/Aβ42 ratio in AD patients in comparison to age-matched control individuals [80]. The study of Fukumoto et al. has indicated that AβOs might be useful in the discrimination between AD/MCI patients and cognitively normal control, thus elevated AβO levels may predict the conversion of MCI to AD [81]. Moreover, high or measurable CSF AβO concentrations correlated with elevated risk of AD [82]. These investigations suggest that higher levels of oligomeric CSF Aβ40 could be a potential biomarker useful in the diagnosis of AD with diagnostic sensitivity and specificity greater than 95% and 90%, respectively [83]. Opposite findings were obtained by other authors, who revealed no significant changes in AβOs levels in AD patients [84, 85]. In conclusion, the studies concerning the usefulness of Aβ oligomers are limited and have been inconsistent due to methodological issues that complicate measurement of Aβ oligomers in CSF, however give hope to improved diagnosis of AD patients [86].

## Tau protein and its phosphorylated forms

Tau proteins belong to the family of microtubule-associated molecules that are found in neuronal and non-neuronal cells. This protein has six isoforms with the lengths range from 352 to 441 amino acid residues [87, 88]. Tau proteins play a role in neuronal microtubule stability, and may be also involved in promoting microtubule nucleation, growth, and bundling. In addition, the studies performed on animal models have revealed that expression of Tau protein reflects the process of neurofibrillary tangle formation, rather than tangles themselves, and is responsible for synapse and neuronal loss. The total Tau protein concentration was proved to be a nonspecific marker of neuronal destruction in neurodegeneration. A meta-analysis investigation has found that all reported studies indicate increased CSF Tau levels in AD patients [89]. Moreover, increased CSF Tau concentrations might be also found in patients with other neuropsychiatric diseases such as CJD [90].

In contrast to Tau protein, being a very sensitive biomarker of neurodegeneration, but unspecific for a particular condition, pTau proteins seem to reflect more specific alterations in Tau metabolism in AD [40]. For example, pTau181 remains unchanged, whereas total Tau is increased, after acute stroke. Tau-microtubule interactions are regulated by the phosphorylation of Tau molecules [91]. Thus, hyperphosphorylated Tau or oligomeric Tau might be involved in synaptic degeneration, while granular Tau oligomers are responsible for neuronal loss [92]. It is suggested that adding the measurements of soluble oligomers of Tau protein (TauOs) to the panel of CSF biomarkers could improve the diagnosis of AD. The toxicity of TauOs seems to be a potential pathogenic factor acting on the initial stages of this disease and may be responsible for seeding Tau pathology within AD brains [93].

Several other mid-domain phosphorylated tau residues, including threonine 181 (pTau181), threonine 231 (pTau231), serine 235 (pTau235), serine 199 (pTau199) [94, 95] as well as for the C-terminal residues serine 396 and 404 [96] have been found. It was proven that CSF levels of pTau181, pTau199 and pTau231 had a similar performance to discriminate AD from other neurodegenerative disorders and non-demented controls [97]. In addition, the CSF concentrations of pTau181 are significantly increased in AD patients with clinical diagnoses neurochemically supported by decreased Aβ42 in the CSF [39], which might suggest that pTau181 is not only a marker of simple neuronal loss. Similar results were presented by other authors, who revealed significantly higher levels of CSF pTau181 in AD patients when compared to patients with frontotemporal degeneration, DLB, Parkinson’s disease (PD) or multiple system atrophy [98, 99]. The study of Parnetti et al. [100] has indicated that pTau181 might be used as a biomarker for distinguishing between AD and DLB. Moreover, phosphorylation of Tau protein at both the threonine 231 and serine 235 positions was proved to be elevated in MCI patients who progress to AD within the follow-up period [101].

Some authors suggest the role of pTau in the prediction of AD development. Buchhave et al. [102] assessed the ability of CSF pTau to predict development to AD within 9–10 years in MCI patients and compared CSF biomarkers between early and late converters to AD. At baseline, pTau concentrations were significantly higher in patients who converted to AD during follow-up compared with nonconverters. Moreover, levels of pTau were significantly elevated in early converters in comparison to late converters, while a baseline Aβ42/p-tau ratio predicted the AD development within 9.2 years with a sensitivity of 88%, specificity of 90%, negative predictive value of 86% and positive predictive value of 91%. The authors conclude that about 90% of MCI patients and pathologic CSF biomarker levels at baseline develop AD within 9–10 years [102].

## Diagnostic-oriented interpretation of the biomarkers pattern: the Erlangen Score

The pattern of decreased CSF Aβ42 concentrations and/or Aβ42/40 ratio, along with elevated CSF levels of Tau and/or pTau, as discussed so far, presented two pathophysiologic processes of AD such as amyloidosis and neurodegeneration. These CSF biomarkers show high diagnostic accuracy, and might be routinely used as an AD diagnostics tool in some countries. However their global acceptance is hampered due to lack of comparability of the results achieved in different laboratories as well as using different analytical methods. This has already been addressed, to some extent, by efforts to standardize procedures for collection of samples, assay calibrators, and measurement protocols, however the global acceptance of these novel approaches will certainly need time [103–105]. In addition, as the AD CSF biomarkers are more often measured in clinical use, interpretation of these results required expertise. The question remains how to interpret the information given by the biomarkers, that is often heterogeneous. Thus, biomarkers in some cases falling into clear-cut normal/abnormal categories. To harmonize the diagnostic-oriented interpretation of the profiles of CSF biomarkers, the Erlangen Score (ES) interpretation algorithm was first proposed [106], followed by other approaches, including logistic regression models [107], classification scales based on the number of pathologic biomarkers, such as the Paris–Lille–Montpellier (PLM) scale [108], or a descriptive, nominal-scale A/T/N system [109].

**Fig. 1:**
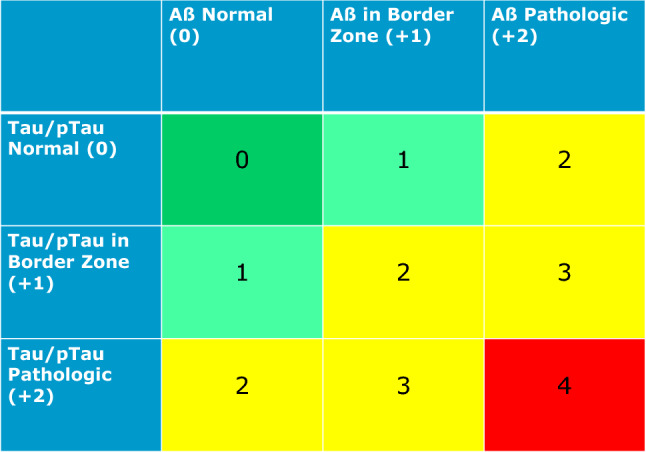
Erlangen Score interpretation algorithm; adapted from Lewczuk et al. [16]

According to the Erlangen Score, result of CSF with all biomarkers normal is scored with 0 points, and interpreted as “no neurochemical evidence for AD”; a pattern with border zone changes in one biomarkers’ group (either Aβ or Tau/pTau, but not both) results in the score of 1, and is reported as “neurochemically improbable AD”; a CSF result with evident alterations in either Aβ metabolism (decreased Aβ42 concentration or Aβ42/Aβ40 ratio) or tau metabolism (increased Tau levels and/or pTau181) but not both is scored 2 points; the same score is given in the case of border zone alterations in the CSF biomarkers of both groups. A result with evident changes in one biomarkers’ group (either Aβ or Tau) accompanied by border zone alterations in the other group is scored three points; these two cases (with the ES = 2 or 3) are interpreted as “neurochemically possible AD”. Evident changes in both Aβ and Tau groups result in four points, and are interpreted as “neurochemically probable AD”. Finally, isolated, very high Tau levels is reported as suspected rapidly progressing neurodegeneration with improbable AD. However, the same Tau levels, together with pathologic Aβ42 concentrations/ratio would shift the interpretation to possible or even probable AD depending (if pTau is normal or not, respectively). The Erlangen Score pattern can be summarized and reported to clinicians in a graphical form, that was presented in Fig. 1.

One of the core concepts in the ES algorithm is the border zone result. They need to be, perhaps arbitrary, defined by each laboratory, with suggested value of about 10%. Irrespectively of the magnitude, however, it is important to note that border zones of the ES are asymmetric; they affect exclusively results on the pathologic side of the reference value. For example, with the reference value of Aβ42/40 ratio in Erlangen laboratory being 0.05, all results above this cut off are considered entirely normal, but results within 0.045–0.05 are interpreted as “borderline pathologic”. Similarly, results of Tau lower than 320 pg/ml are considered normal, but results within 320–350 pg/ml are considered borderline pathologic. Such asymmetric distribution of the interpretation has ethical reasons; in the situation of lacking of effective treatment, we believe it is ethically more correct to underdiagnose patients, by accepting higher ratio of false negative errors than to overdiagnose (by making more false positive errors). Of course, as soon as effective and safe treatment appears on the market, the concept of interpretation of the border zone results will have to be reconsidered.

This concept shows clear advantages compared to other approaches. It enables categorization of the CSF results into five classes on an ordinal scale (0–4), with increasing degree of changes of the CSF biomarkers in AD. Further, it includes, for the first time in the interpretation of the AD biomarkers in CSF, the idea of border zone results. ES improves precise stratification of patients into five categories with increasing degree of the CSF pathology, opposite to a dichotomous approach (CSF normal/pathologic) applied earlier [42]. In addition, ES is less complicate in comparison to regression-based approaches [107]; in every-day laboratory routine it does not require computer-based support at all, although it is easily coded to provide high-throughput laboratories with automated computer-supported lab systems. When we compared the ES to the A/T/N classification [109], this pattern stratifies individuals into classes on an ordinal scale, and not into entirely descriptive categories, that enables at least semi-quantitative correlation of the CSF findings with other metrics, such as odds ratios, progression hazards, or time to progression from MCI to dementia. Further, as an ordinal-scale classification system, the ES provides to take border zone laboratory results into consideration, easily incorporating them into the interpreting algorithm. Finally, ES is more adjustable, enabling inclusion of further potential biomarkers (as long as they reflect amyloid pathology or neurodegeneration at least on an ordinal scale) without necessity to recalculate the ranges (i.e., the number of categories) in comparison to the PLM approach, based on the number of the pathologic CSF biomarkers [108]. Irrespectively of the number of the biomarkers considered, the ES will always classify the CSF patterns into five ordinal categories.

ES has been extensively validated based on cohorts from different expert centers, and on the ground of a broad spectrum of predefined end points. First, ES was proved to correctly classify MCI individuals at increased risk of developing dementia in two independent, large-scale, multicenter cohorts (German Competence Network Dementias, and US-ADNI), irrespectively of the fact that the two projects used entirely different sample handling protocols, uncorrelated center-specific reference ranges and disparate laboratory analytical platforms [110].

In another study, it turned out that MCI subjects classified as “neurochemically probable AD” presented 8–12 times elevated hazards to develop dementia when compared to those classified as “neurochemically improbable AD”, when adjusted for gender, age, MMSE score as well as genotype of APOE. These hazard ratios were clearly time-independent. On contrary, the hazards involved with the cognitive, demographic, and genetic confounders were fully explained by the Erlangen Score [111].

With neuropsychologic and neuroimaging outcome as the end points, it was found that a higher ES predicted a faster disease progression in MCI patients; the subjects with higher ES showed a faster reduction of the whole brain and the hippocampal volumes, as well as faster decrease in MMSE, and a faster increase in ADAS-Cog scores [112].

Finally, Erlangen Score algorithm is enabled to correct prediction of the post-mortem neuropathological outcome on the ground of the intra vitam CSF results of three core AD biomarkers. The probabilities to have AD pathology post mortem in contrast to non-AD pathologies including mainly FTLD, VaD, and LBD increased almost linearly with increasing ES ordered categories, with less than 3% of the neuropathologically definite AD patients (3 out of 106) were categorized as neurochemically improbable AD (ES = 0 or 1) [113].

## Novel candidate biomarkers for AD

A number of CSF biomarkers specific for pathologic changes, as well as those unspecific markers of oxidative damage or inflammation in AD have been tested, but many of them have only been reported in single studies [114]. Several novel potential candidate biomarkers (summarized in Table 1) have been proposed and investigated, however most of them have not been validated and integrated into diagnostic guidelines [30].

**Table 1:** Potential significance of novel biomarkers of AD

Pathophysiological mechanism	Fluid biomarker	Alterations in AD	References
Aβ metabolism	β-site amyloid precursor protein cleaving enzyme 1 (BACE1)	Increased levels/activity in CSF and increased activity in plasma	[116–118]
Vascular dysregulation	Heart-type fatty acid-binding protein (hFABP)	Consistently increased in CSF, but no change in plasma or serum	[119, 120]
Inflammation/glial activation	Triggering receptor expressed on myeloid cells 2 (TREM2) and its soluble variant (sTREM2)	Increased in CSF, but no change in plasma levels	[121, 122]
	Chitinase-3-like protein 1 (YKL-40)	Increased in CSF and plasma	[123, 124]
	Interferon-γ-induced protein 10 (IP-10)	Inconsistent results in CSF, plasma or serum	[125]
	Matrix metalloproteinase-9 and -3 (MMP-9 and MMP-3)	Increased in CSF MMP-3 Decreased in CSF MMP-9	[136]
Synaptic dysfunction	Neurogranin	Increased in CSF, but no change in plasma; Increased CSF Neurogranin seems to be specific for AD	[126, 127]
	Synaptosome-associated protein 25 (SNAP‑25)	Increased in CSF	[128]
	Synaptotagmin-1 (SYT-1)	Increased in CSF	[129]
α-Synuclein pathology	α-Synuclein	Increased in CSF	[130, 131]
TDP-43 pathology	TDP-43	Increased in plasma	[132]
Iron metabolism	Ferritin	Increased in CSF levels is associated with cognitive decline, no changes in plasma	[133]
Neuronal proteins	Visinin-like protein 1 (VILIP-1)	Increased in CSF and plasma	[134, 135]
	Neurofilament light (NF-L)	Consistently increased in CSF and increased in plasma; May serve as simple, noninvasive screening tool	[137, 138, 159]

Novel fluid biomarkers were grouped, based on the corresponding pathophysiologic AD mechanism, including Aβ metabolism (Aβ38, AβOs, BACE1), vascular dysregulation (heart-type fatty acid-binding protein—hFABP), inflammation and glial activation (triggering receptor expressed on myeloid cells 2—TREM2 and its soluble variant—sTREM2, chitinase-3-like protein 1—YKL-40, interferon-γ-induced protein 10—IP10), synaptic dysfunction (neurogranin, synaptosome-associated protein 25—SNAP‑25 and synaptotagmin-1—SYT-1), α-Synulcein pathology (α-synuclein), TDP-43 pathology (TAR DNA binding protein 43—TDP-43), iron toxity (ferritin) and other neuronal proteins (visinin-like protein 1—VILIP-1 and neurofilament light—NFL) [115–138]. Currently, many other candidates for AD biomarkers are under intensive investigations, including selected metalloproteinases (MMPs) and their tissue inhibitors [136]. For example, CSF concentrations of MMP-9 are significantly lower, while MMP-3 levels are significantly elevated in AD patients in comparison to the elderly individuals without cognitive deficits. In addition, MMP-2, TIMP-1 and TIMP-2 show no significant changes among the groups investigated (AD, MCI patients and elderly individuals without cognitive deficits). Taken together, this might indicate that these proteins play a potential role in the pathophysiology of AD and diagnostics, however other studies performed on larger group of patients need to be performed to establish their potential diagnostic utility [136].

Although changes in the metabolism of Aβ are currently considered the earliest detectable events in AD, interventional strategies based on the Aβ hypothesis are still disappointing [139, 140]. This calls for more extensive researches of other hypotheses, of which those related to Tau seem particularly attractive [141]. This is further supported by the observation that cognitive symptoms in AD are in direct relation to the biomarkers of neurodegeneration rather than to Aβ deposition biomarkers. In neuropathological investigations, a clear correlation was indicated between the degree of post-mortem neurofibrillary tangle pathology and a patient's cognitive functions intra vitam [142, 143]. Following this line of argumentation, a novel assay capable to specifically measure non-phosphorylated forms of Tau molecules (Non-P-Tau) was recently developed [144]. Interestingly, it significantly improved the proportion of correctly classified patients (99%) compared to that achieved by the assays used routinely so far: Tau (90%), pTau181 (62%) and 14-3-3 (91%) [145]. Furthermore, Non-P-Tau assay is extensively used in differential diagnosis of other dementias, particularly those with profound Tau pathology (like FTLD), although final conclusions have not been reached yet [146].

Another potentially interesting candidate in AD differential diagnosis might be α Synuclein [131, 147]. A vast majority of studies published so far reports reduced CSF levels of total αSyn in PD subjects, in comparison to both healthy controls and/or patients with other neuropsychiatric disorders [148]. On the other hand, large proportion of papers shows that CSF total αSyn concentrations tend to increase in AD compared to the controls [149]. Increased αSyn concentrations in the AD subjects, along with moderate correlation between αSyn and Tau/pTau181 in the current study reconfirm the data and the conclusions reported by many investigators, that the increased CSF αSyn in AD seems to rather reflect unspecific neurodegeneration and not specific process characteristic for AD [150, 151]. Indeed, considering that predominant source of αSyn in the brain is presynaptic neuronal terminals, it seems reasonable to hypothesize that degenerating neurons passively release αSyn molecules, which then diffuse to the CSF at increased rate.

Further investigations are also needed to clarify association between the biomarkers and clinical presentation such as cognitive measures, and the effects of patient variables such as sex, APOEε4 status, comorbidities.

## Novel analytical platforms in biomarkers’ research

Development of novel technologies for biomarkers’ research and diagnostic applications has faced the most rapid progress in the last decades [152]. Driven by the needs to improve the quality of performance of already established biomarkers, and to enhance analytical sensitivity to search for novel candidates (like those in the blood, for example), new technological platforms have been developed since the first methods to analyze AD biomarkers in the CSF (ELISA and Western blot) were established back in 1990’s.

A mass-spectrometry proteomic is often used in search for novel biomarkers. Its advantage relays mostly on the fact that it can directly analyze the molecule in question; the drawback, however, is that mass spectrometry is usually less effective, compared to immunoassays, in the analysis of intacted larger molecules. To overcome this limitation, approaches have been developed that combine advantages of mass spectrometry with ligand-based techniques. As a result, enzyme-linked immuno mass spectrometry assays have been developed [153] that provide improved analytical sensitivity compared to conventional ELISA.

Another issue in AD biomarker development and their clinical application relates to sample volume limitation. Currently, CSF is regarded as the exclusive source of the fluid-based AD biomarkers (although the first blood-based biomarkers seem appear on the horizon); as a consequence, laboratories need to overcome limitation in availability of the diagnostic and/or human research material. To overcome this issue, multiplexing technologies have been developed that enable simultaneous quantification of several biomolecules in a single, minute sample volume [154, 155]. Advantage of multiplexing is that it analyzes all relevant biomarkers in exactly the same sample and under exactly the same conditions. This is of crucial importance since eventual interpretation of the results derived from the CSF, not only in case of neurodegeneration but even more strongly in neuroinflammation, relays on a pattern of several biomarkers and not on unidimensional result of a single analyte [156]. The drawback of multiplexing, however, is that it is difficult to find such set of antibodies that would be sensitive enough to capture molecules of interest and simultaneously would not cross-react with one another. Similarly, all analytical reactions must take place under the same conditions (dilution buffer, incubation temperature and time), and hence these conditions cannot be optimized for every reaction but must be a compromise across them. Particularly, all the molecules of interest must fit approximately the same range of concentration due to the fact that all of them are, by definition, prediluted to the same degree with the assay buffer [157].

A Single-Molecule Array (Simoa®) has been developed by a company Quanterix, as a combination of generation of ELISA-like sandwich complexes which, after formation on magnetic beads are subsequently transferred onto an array of microwells, and sophisticated statistical software capable to interpret the signal in a different manner depending on the number of signal-emitting beads per microwell [158]. Application of this ultrasensitive technology seems to be particularly relevant for low-concentration candidate biomarkers in the blood [159].

Another interesting concept is the application of the polymerase chain reaction (PCR) property to amplify a single DNA copy to many orders of magnitude for development of ultrasensitive AD biomarkers immunoassays. With this technology, molecules are captured between magnetic beads coated with antibodies modified with DNA molecules that are subsequently amplified to generate strong signal [160].

To finish this short technology overview, a technology of immuno-magnetic reduction developed by a company MagQu is perhaps worth mentioning. Briefly, analytes are captured by magnetic nanoparticles coated with specific antibodies, which lead to a concentration-dependent decrease of the oscillations of the nanoparticles in alternating magnetic field. Advantage of this technology relays, among other, on application of only one antibody, in contrast to majority of other platforms, which use two [161].

## Conclusion

AD is age-related neurodegenerative disease characterized by progressive synaptic damage and neuronal loss. The disease affects a substantial proportion of elderly. The cause of AD remains still unresolved. The hallmark pathological features of AD are senile plaques composed of Aβ and neurofibrillary tangles (NFTs) of Tau protein within patients’ brain. Four biomarkers in the CSF (Aβ42, Aβ42/40, Tau, and pTau181) have been extensively studied and validated as core AD biomarkers. Fluid biochemical markers measured in CSF hold promise for enabling more effective drug development and establishing a more personalized medicine approach for AD diagnosis and treatment. Despite large gains in our understanding of AD pathogenesis, validated biomarkers for early detection and accurate diagnosis are sorely needed.
